# *Mycobacterium avium* subsp. *paratuberculosis* in Wild Boar (*Sus scrofa*) in Portugal

**DOI:** 10.3390/pathogens13050389

**Published:** 2024-05-08

**Authors:** Ana Cristina Matos, Luis Figueira, Maria Helena Martins, Luís Cardoso, Manuela Matos, Maria de Lurdes Pinto, Ana Cláudia Coelho

**Affiliations:** 1 Polytechnic Institute of Castelo Branco, 6001-909 Castelo Branco, Portugal; acmatos@ipcb.pt (A.C.M.); lmftfigueira@gmail.com (L.F.); lena@ipcb.pt (M.H.M.); 2 Research Center for Natural Resources, Environment and Society, Polytechnic Institute of Castelo Branco, 6001-909 Castelo Branco, Portugal; 3 Quality of Life in the Rural World (Q-RURAL), Polytechnic Institute of Castelo Branco, 6001-909 Castelo Branco, Portugal; 4 Animal and Veterinary Research Centre (CECAV), Department of Veterinary Sciences, University of Trás-os-Montes e Alto Douro (UTAD), 5000-801 Vila Real, Portugal; lpinto@utad.pt (M.d.L.P.); accoelho@utad.pt (A.C.C.); 5 Associate Laboratory for Animal and Veterinary Sciences (AL4AnimalS), 5000-801 Vila Real, Portugal; 6 Centre for the Research and Technology of Agro-Environmental and Biological Sciences (CITAB), University of Trás-os-Montes e Alto Douro (UTAD), 5000-801 Vila Real, Portugal; mmatos@utad.pt

**Keywords:** epidemiology, *Mycobacterium avium* subsp. *paratuberculosis*, wild boar

## Abstract

Paratuberculosis, or Johne’s disease, caused by *Mycobacterium avium* subsp. *paratuberculosis* (MAP), is a chronic granulomatous enteritis affecting both domestic and wild ruminants. The agent was also found in wild mammals such as wild boar (*Sus scrofa*); however, the role of wild mammals in the epidemiology of MAP is unclear. During the research period, 941 free-ranging wild boar (*S. scrofa*) legally hunted in two locations in the central–eastern region of Portugal were examined. Ninety-seven wild boars exhibited one or more gross lesions and were tested for the presence of *Mycobacterium avium* subsp. *paratuberculosis* using acid-fast staining, mycobacterial culture, polymerase chain reaction (PCR), and histopathological examination. Forty-five animals (46.4%, 95% CI: 36.5–56.3%) were identified as infected, as indicated by positive results in culture and/or PCR. The findings revealed that the most significant risk factor was being a juvenile compared to yearlings and adults (OR = 10.2, 95% CI: 2.2–48.0). Based on our results, 37.9% (*n* = 11) of the infected animals were considered suitable for human consumption. Our findings offer novel insights into mycobacterial infections in wild boar populations in Portugal and suggest that wild boar could be a source of human infection if zoonotic potential is considered.

## 1. Introduction

Domestic ruminants are naturally susceptible to *Mycobacterium avium* subsp. *paratuberculosis* (MAP), which is the causative agent of chronic granulomatous enteritis, known as paratuberculosis or Johne’s disease [1,2].

MAP has been isolated in different free-living ruminant species, such as red deer (*Cervus elaphus*), fallow deer (*Dama dama*), and roe deer (*Capreolus capreolus*), which grazed on pastures that were simultaneously or previously used for domestic ruminants [3,4,5], as well as in non-ruminant wildlife, such as wild boar (*Sus scrofa*) [3,6,7], red fox (*Vulpes vulpes*) [8,9,10], Eurasian otter (*Lutra lutra*), European badger (*Meles meles*) [10,11], Egyptian mongoose (*Herpestes ichneumon*) [10], and wild rabbits (*Oryctolagus cuniculus*) [12]. However, the role of wild mammals in the epidemiology of MAP is unclear because these species do not usually exhibit the classical clinical signs of paratuberculosis [3,6,7,10]. Many factors can influence the prevalence and spread of paratuberculosis in domestic animals, such as failure in biosecurity measures, farm management practices, and contact between wildlife and livestock [3,13,14,15,16].

Wild boar has been a common species in Portugal since the second half of the 20th century [17]. This expansion has led to various implications, especially concerning infectious diseases and zoonotic risks associated with wild boar. Studies have identified wild boar as potential reservoirs for infectious agents like HEV, *Coxiella*, and *Brucella* [18]. The presence of these pathogens in wild boar poses risks not only to wildlife but also to domestic animals and humans due to potential zoonotic transmission. The adaptability of wild boar to different habitats and food resources contributes to their continuous occupation of new geographic regions, further necessitating comprehensive infectious disease assessments. It is crucial to understand the prevalence of pathogens in wild boar for infectious disease surveillance, disease management strategies, and wildlife conservation efforts in Portugal [18].

Large-scale surveys to identify the presence of MAP in wild mammals are still limited in Portugal, according to previous studies [4,7,10,19,20,21,22,23,24,25]. Since it was first reported in Portugal in 1983, there have only been a few surveys organized to estimate paratuberculosis infection in domestic animals [22,23,24,25].

The detection of bacteria by culture or molecular methods and the evaluation of histopathological lesions constitute the most accurate methods of diagnosis for paratuberculosis [26]. PCR assay is one of the most up-to-date methods used for various purposes since it can detect small amounts of DNA and operates more rapidly compared to other techniques [24]. Public health concerns about the presence of MAP in food, such as meat and milk, are increasing [27,28] due to accumulating data that link MAP to human Crohn’s disease (CD), a chronic, incurable, low-grade inflammation of the terminal ileum [28,29,30,31,32,33,34,35,36]. To date, studies have shown that MAP is also implicated in type 1 diabetes mellitus [35,36] and in Blau syndrome tissues [37]. Milk and dairy items are recognized as the main avenue for MAP infection in humans. Even pasteurized milk products pose a consumption risk since pasteurization merely decreases the original MAP load in milk [38,39]. MAP was identified in yogurt [40], cheese [41], muscle meat [42], and hamburger [43]. Investigating the presence of MAP is essential to manage the spread of this pathogen in wild boar. By identifying the prevalence of MAP, authorities can implement specific control measures to prevent its transmission among wildlife and livestock. Moreover, identifying risk factors can help mitigate the risks associated with MAP infection in both wildlife and human populations. This study aimed to investigate the prevalence of MAP in wild boar and identify associated risk factors.

## 2. Materials and Methods

### 2.1. Animals and Samples

This study analyzed 941 wild boar (*S. scrofa*) that were legally hunted in the cities of Idanha-a-Nova (39°55′11″ N, 7°14′12″ W) and Penamacor (40°10′8″ N, 7°10′14″ W), located in Castelo Branco, in the eastern–central region of Portugal, during the period of 2010 to 2022. All animals were thoroughly examined by a qualified veterinarian during sanitary inspection after hunting. For the animals that displayed any visible gross lesions or had clinical signs such as weight loss or a rough coat (*n* = 97), multiple tissue samples were collected and analyzed using acid-fast staining, mycobacterial culture, polymerase chain reaction (PCR), and histopathological examination. The samples collected at the post-mortem examination included retropharyngeal, mediastinal, and bronchial lymph nodes, as well as mesenteric lymph nodes, palatine tonsil, lung, liver, spleen, kidney, ileocecal valve, distal jejunum, and ileum. The collected tissues were processed using standard techniques to prevent cross-contamination between samples and animals. Subsequently, the tissues were divided into three portions: two were promptly frozen and preserved at −80 °C for PCR assays and mycobacterial culture, while the third portion was immediately fixed in 10% neutral buffered formalin. During the collection process, relevant data regarding the animals were recorded including the date, location, estimated age, sex, and body condition. To determine age, tooth eruption and replacement patterns were evaluated. Animals less than 12 months old were classified as juveniles, while those between 12 and 24 months old were classified as yearlings, and those over 2 years of age were classified as adults [44].

### 2.2. Pathological Examination

The pathological examination involved a comprehensive necropsy, encompassing a detailed macroscopic inspection of the retropharyngeal, submandibular, and parotid lymph nodes in the head; tracheobronchial and mediastinal lymph nodes; and the lungs in the thorax. In the abdominal region, a thorough examination was conducted on the hepatic, mesenteric, and ileocecal lymph nodes, along with the ileocecal valve, liver, kidneys, and spleen. Additional gross lesions were noted in other areas. Lymph nodes were carefully examined and sliced into sections. Lesions in organs that were observed during a more thorough laboratory examination were considered to determine the presence of gross lesions. Tissue samples were imprinted and stained with the Ziehl–Neelsen (Z-N) method to identify acid-fast rods (AFRs). At least 100 different fields were examined using an oil immersion objective (100×) in each sample. The tissue samples were fixed using a 10% neutral buffered formol saline solution by immersion and then processed for histopathology using routine techniques for paraffin embedding. The tissue sections were sectioned at 4 μm, and then stained with hematoxylin and eosin (HE) and the Ziehl–Neelsen (Z-N) and Gram staining techniques. Histopathological lesions with regard to the type of inflammatory infiltrate and the presence of acid-fast organisms were observed and recorded.

### 2.3. Tissue Culture

Culture methodology was conducted, as described previously [45,46]. Briefly, five culture media were used and incubated at 37 °C for 6–12 months. The five media used in the study were the Löwenstein–Jensen medium (Liofilchem, Roseto degli Abruzzi (TE), Italy), Löwenstein–Jensen medium with mycobactin J (Synbiotics Europe SAS, Lyon, France), Löwenstein–Jensen medium with sodium pyruvate without glycerol, Middlebrook 7H11 medium supplemented with OADC (oleic acid-albumin-dextrose-catalase) (Becton Dickinson, Franklin Lakes, NJ, USA), and Middlebrook 7H11 medium supplemented with OADC and sodium pyruvate without glycerol. Samples were decontaminated with 0.75% (*w*/*v*) hexadecyl pyridinium chloride (HPC; Sigma-Aldrich, Milan (MI), Italy) for 18 h, and cultured in duplicate, using five specific media for mycobacteria, supplemented with a mix of amphotericin B (50 mg/L), penicillin (100,000 U/L), and chloramphenicol (100 mg/L) (Sigma-Aldrich, Milan (MI), Italy). Colonies with compatible mycobacterial morphology were tested for acid-fastness bacilli using the Z-N staining method. The mycobacterial isolates were tested for MAP confirmation using the PCR as mentioned above methods. Acid-fast mycobacteria that tested negative for MAP by PCR were further examined to determine their identity using PCR amplification of the 16SrDNA gene described below [47].

### 2.4. DNA Extraction

According to manufacturer instructions, genomic DNA was extracted from tissues, and MAP culture isolates were extracted with a commercial DNA preparation kit (DNeasy Blood and Tissue Kit, Qiagen, Hilden, Germany). Samples were stored at −20 °C until used as the template in PCR assays. DNA from bacteria isolated on tissue culture was extracted by taking a loop-full of a culture of Löwenstein–Jensen containing mycobactin, and then transferred to a microcentrifuge vial containing 100 mL 10 mM Tris-HCl/Triton X-100 1%/1 mM EDTA (TTE) and incubated for 20 min at a temperature of 95 °C. After centrifugation, the supernatant was stored at −20 °C until used.

### 2.5. Polymerase Chain Reaction (PCR)

The identification of MAP was confirmed by PCR using IS900 primers. DNA from tissues and bacteria was tested in duplicate for MAP using primers RJ1 (GTT CGG GGC CGT CGCTTA GG) and PT91 (CCC ACG TGA CCT CGC CTC CA) to amplify a 389 bp product [24,48]. The PCR mix consisted of 3 µL DNA, 1 µL of each primer (10 µM), 10 µL Taq-PCR master mix (Qiagen, Hilden, Germany), and 5 µL ultra-pure distilled water (Qiagen, Hilden, Germany) in a final volume of 20 µL.

Amplification was achieved using the following conditions: 2 min at 96 °C, followed by 40 cycles of 30 s at 95 °C, 30 s at 55 °C, and 1 min at 72 °C, and a final 10 min extension at 72 °C. Samples of 20 µL PCR products were analyzed on 1.0% agarose gels running at 90 V for 1 hr. The gels were stained using ethidium bromide. In addition to the samples, a positive (MAP DNA) and a negative (water) preparation control as well as a blank control were included.

Samples that tested negative for MAP by PCR were tested initially by a modified PCR for 16S rDNA reaction, as described by Moravkova et al. [47]. This assay allows for the identification of DNA from bacteria from the genus *Mycobacterium* and the differentiation between *M. avium* and *M. intracellulare* other atypical mycobacteria. The PCR amplification reaction was performed in a total volume of 20 mL containing 2 mL of isolated DNA, 1 mL of each primer (10 mM), and 10 mL of Taq PCR Master Mix (Qiagen, Hilden, Germany). Specific primers used for this assay were MYCGEN-F (50-AGAGTT TGA TCC TGG CTC AG-30), MYCGEN-R (50-TGC ACA CAG GCC ACA AGG GA-30), MYCAV-R (50-ACC AGA AGA CAT GCG TCT TG-30), and MYCINT-F (50-CCT TTA GGC GCA TGT CTT TA-30).

A negative control (sterile water) and a positive control DNA from *M. avium* subsp. *paratuberculosis* strain ATCC19698 were included in each amplification run. An amplification product of 1030 bp is indicative of the genus *Mycobacterium*, an 850 bp fragment for *M. intracellulare*, and a fragment of 180 bp is positive for *M. avium*.

### 2.6. Statistical Analysis

The association between the clinical and pathological parameters and the infection status was analyzed. The animals were classified as infected if MAP was isolated in the organs by culture and/or detected by PCR in tissue samples, and as uninfected if no MAP was isolated or detected. To compare the effectiveness of culture and PCR in granulomatous lymphadenitis, the Cohen’s Kappa (K) coefficient was used to measure the proportional agreement [49] on the results obtained in the different laboratory methods. K values indicate the following: 0.01–0.20, slight agreement; 0.21–0.40, fair agreement; 0.41–0.60, moderate agreement; and 0.61–0.80, substantial agreement. A value of K > 0.80 represents excellent non-random proportional agreement. The program IDoStatistics^®^ (https://idostatistics.com/cohen-kappa-free-calculator/#risultati, accessed on 1 April 2024) was used for this calculation.

For statistical analysis, the three age classes were transformed into two age classes: one class of juveniles and yearlings and the other of adult subjects. The infection status was analyzed using contingency tables and a Chi-square test (two-sided). Test statistics were considered significant at *p* < 0.05. Univariate analysis was carried out using Chi-square test analysis. Confidence limits for the proportions were established by the exact binomial test with a 95% confidence interval (CI). All statistical analyses were conducted using the statistical software package SPSS^®^ 25.0.

## 3. Results

Out of the 941 wild boar (*Sus scrofa*) that were hunted and examined, 97 showed one or more gross lesions and were further studied by microbiological and pathological methods. Among the animals analyzed, 49 (50.5%) were female and 48 were male. A total of 15 (15.5%) animals were juveniles, 38 (39.2%) were yearlings, and 44 (45.4%) were adults. The mean age of the animals for which we have age estimates was 2.3 years, and the oldest was 6.

PCR assays revealed DNA from the *Mycobacterium* genus in 35 (36.1%) of the 97 animals. These were identified as *M. avium* in 32 (32.9%) animals, detected by 16S rDNA PCR. IS900 PCR detected *M. avium* subsp. *paratuberculosis* in 30 (30.9%) mesenteric lymph nodes and was also positive in three (3.1%) retropharyngeal lymph nodes and in eight (8.2%) kidneys.

MAP was isolated by culture from 21 (21.6%) of the 97 animals. The isolates were found in mesenteric lymph nodes, the intestinal and pulmonary lymph nodes, intestinal mucosa, and kidney.

In the 28 mesenteric lymph nodes with lesions reported as granulomatous lymphadenitis, the presence of lymphocyte cells (*n* = 27; 96.4%) and caseation necrosis (*n* = 22; 78.6%) were the most common features. Lesions were always multifocal and ranged from occasional proliferative lesions, less than 1 cm in diameter (71.4%), of necrotic granulomas to large areas of granulomatous lesions, more than 1 cm (28.6%) in diameter, and of either necrotic or necrotic calcified granulomas.

The calcification area was smaller or similar to the necrotic area. The granulomas were typically composed of a necrotic core, surrounded by a population of lymphocytes, plasma cells, macrophages, and epithelioid macrophages, frequently separated from the normal parenchyma by a peripheral fibrous capsule. Multinucleated giant cells, which occurred in 21.4% of the cases, were always of the Langhans type. In most of the lesions (24.7%), the necrotic core was formed by caseated material, but in many cases (16.5%), liquefactive necrosis was also present at the center of the granulomatous lesion. In those cases, bacterial colonies (non-mycobacterial) and neutrophils (12.4%) were observed within the core and the surrounding inflammatory cell population. A small percentage of cases (7.1%) showed exclusively liquefactive necrosis at the center of the lesion. Of the 28 lymph nodes with histopathological diagnosis of granulomatous lymphadenitis, 15 (53.6%) were PCR negative and 22 (78.6%) culture negative. One specimen was PCR-negative despite its culture positivity. The culture was negative in eight PCR-positive specimens. The value of Cohen’s k was 0.329 and the % of agreement was 67.9%, which is a fair agreement (0.21–0.40) (Table 1).

**Figure 1:**
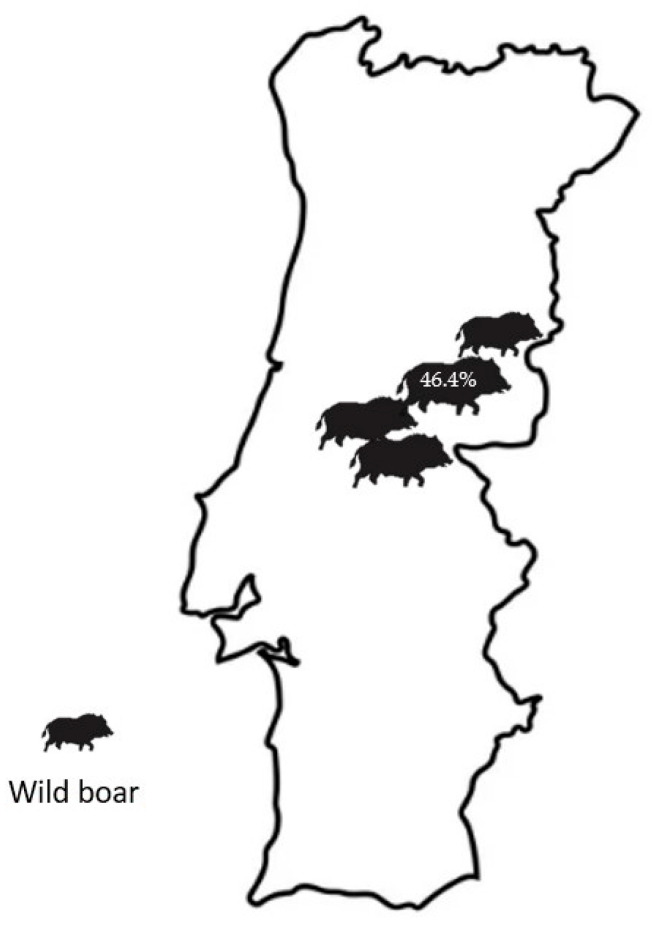
Prevalence of *Mycobacterium avium* subsp. *paratuberculosis* in eastern–central Portugal during the hunting season.

Forty-five animals (46.4%, 95% CI: 36.5–56.3%) were classified as infected, as indicated by the positivity in culture and/or in PCR. Of these animals, 3 (6.7%) were hunted in 2009, 29 (64.4%) in 2010, and 13 (28.9%) in 2011. MAP was isolated and/or detected from wild boar in one of the two study areas (Idanha-a-Nova) (Figure 1).

Infection rates for age classes were 28.9% in juveniles, 35.6% in yearlings, and 35.6% in adults. This difference was statistically significant (*p* = 0.001). Infection rates were 57.8% for males and 42.2% for females. This difference, however, was not statistically significant (*p* = 0.129). Of the 33 lymph nodes that tested positive on Z-N smears, 22 (66.7%) belonged to infected animals.

In total, 22 infected animals (48.9%) had lesions in mesenteric lymph nodes, 2 (4.4%) had lesions in the ileocecal valve, 12 (26.7%) in the lungs, 7 (15.6%) showed lesions in mediastinal lymph nodes, 26 (57.8%) in the retropharyngeal nodes, 4 (8.9%) in the intestine, and 3 (6.7%) in the kidneys.

In total, 29 (29.9%) of the wild boar studied were suitable for human consumption, while 68 (70.1%) were unsuitable (rejected) after sanitary inspection of their carcasses. According to our results, 37.9% (*n* = 11) of infected animals were suitable for human consumption.

The univariate analysis associated four variables (*p* < 0.05) with infection. Table 2 shows the odds ratio and 95% confidence interval of those posing a potential risk and predictive factors associated with infection on a factor-by-factor basis, calculated for the variables. These variables included: “Age”; “Lesions in lungs”; “Lesions in mesenteric lymph nodes” and “Positive Ziehl–Neelsen smear in mesenteric lymph nodes”.

The results showed that the strongest risk factor was being a juvenile compared to yearlings and adults (OR = 10.16, 95% CI: 2.15–48.03). Another factor significantly related to increased odds for infection was the presence of lesions in the lungs, being higher in those having lesions when compared with animals without lesions (OR = 3.42, 95% CI 1.19–10.63). The presence of lesions in mesenteric lymph nodes also increased the risk of being infected (OR = 4.02, 95% CI: 1.63–9.92). In this study, those animals that had positive results in Ziehl–Neelsen smears (OR = 3.57, 95% CI: 1.47–8.65) displayed a higher probability of being considered infected.

## 4. Discussion

Wild boar can serve as significant reservoirs for MAP, which can lead to the spread of the disease in other wildlife populations and domestic livestock. Understanding the impact of wild boar in the disease transmission cycle is essential for effective disease control measures. Additionally, studying MAP in wild boar can provide valuable insights into the ecology and evolution of the bacteria, potentially leading to the development of improved diagnostic tools and more effective control strategies.

Few studies have been performed to investigate MAP in wild boar [7,21,50,51,52]. In the present study, we demonstrated that MAP was widespread in Central Portugal. Álvarez et al. [50] reported a 1.5% prevalence of paratuberculosis in wild boar in southern and western Spain, with similar environmental and host population characteristics, considerably lower than the MAP infection rate estimated in the present study. In a previous epidemiological study performed in Korean wild boar in 197 serum and 180 fecal samples, 2 MAP colonies were recovered from 180 fecal samples cultured, 18 animals were positive in PCR, and 1 serum sample had a strong humoral response to MAP [51].

Wildlife diseases continue to pose a major threat worldwide, particularly as they impact the health of humans, livestock and highly valued wildlife populations [53]. Wild boar are a significant concern due to their large migratory range and rapid population growth. They consume vegetables, small vertebrates, young hares, pheasants, and roe deer, as well as the carcasses of free-range and wild mammals that may be infected with causative agents of serious mycobacterial and other diseases [54,55].

Wild boar has been identified as carriers of MAP, posing a significant risk for the transmission of mycobacterial infections to domestic livestock and other wildlife populations. Studies conducted worldwide have highlighted that these infections are most prevalent in areas where ruminants and domestic pigs have previously grazed. Additionally, wild boar can potentially act as a source of mycobacteria for other wild animals [2,51,54,55].

The environment is extensively contaminated by feces from ruminants infected with MAP. The agent is capable of withstanding various physical conditions and can survive for long periods of time in the environment. As a result, it is most likely that infection occurs from wildlife to cattle through indirect contact via the fecal–oral route, with contaminated pasture serving as the source of infection [56].

Based on the number of wild boars examined and the number of infected animals, it is possible that the infection is widespread and may be related to the high density of wild boar in the research area [57]. Wild boar populations in the Iberian Peninsula are reported to range from 1.7 to 12.5 animals per 100 hectares [57,58].

There are several possible reasons why samples tested positive in one test and not in others, such as the limited distribution of focal lesions, the low detection limit of the culture methods employed, the presence of a low number of small-sized lesions, the heterogeneous distribution of the organisms [59], and losses caused by the decontamination procedures used on tissue samples [60]. The existence of paucibacillary paratuberculosis cases, which are characterized by the presence of a small number of organisms in infected tissues or feces, are another possible explanation [61]. However, in this study, well-established culture protocols and decontamination methods [45,46,59] were used, and spiked samples were included as appropriate controls. The use of five different media simultaneously has enhanced the specificity of MAP isolation in culture, enabling a complementary approach to detect various species within the *M. avium* complex. Furthermore, these media facilitate the exclusion of certain non-target species, thereby refining the detection process [26,46].

In this study, age has been identified as the major risk factor for higher infection rates. The data suggest that juveniles are more prone to infections compared to other age groups. The reason behind this age-related resistance is not yet understood. However, it is believed to be linked to the age-related reduction in the surface area and lymphoid follicle density of Peyer’s patches in the small intestine, which has been observed in sheep and cattle. Moreover, changes in the lymphoid cell types, particularly M-cells, dendritic cells, and lymphocytes, may also play a role in age-related resistance [62].

The risk of infection was significantly higher in animals with lesions in the lungs and mesenteric lymph nodes, suggesting that the respiratory and alimentary routes of infection both occur in nature. Slaughter hygiene is crucial due to the high prevalence of MAP in the mesenteric lymph nodes of wild boar. The lymph nodes should be removed to prevent cross-contamination with other parts of the carcass and other carcasses.

The histopathological parameters observed were consistent with previous reviews by Álvarez et al. [50] and Machackova et al. [54]. Our results suggest that smears stained with Ziehl–Neelsen are good indicators of infection and could be used to screen tissue for the presence of typical organisms, which could be confirmed by other methods with the advantage of being cheap and fast. In some cases, acid-fast bacilli were microscopically detected in tissue, but the organism could not be detected by tissue PCR and/or culture. Failure to detect MAP from tissues where numerous acid-fast bacilli were observed by the Ziehl–Neelsen technique may be attributed to non-mycobacterial positive ZN staining, possibly derived from bacteria of the Nocardia genus that are also visible as acid-fast bacilli [63].

A previous study showed that PCR examination of suspected tissue is the best way to confirm a prior diagnosis of MAP infection in wildlife [64]. Our results corroborated this previous report that PCR is the most reliable technique for identifying infected animals. PCR is highly sensitive and specific, capable of detecting even small amounts of target DNA in a sample. This sensitivity is crucial for detection when low concentrations of MAP are present in clinical samples. PCR-based methods are relatively rapid compared to culture-based techniques, which can take weeks to yield results [24,26,48].

This study can have some limitations. The study design may have introduced a potential selection bias, as the samples were obtained only from animals that displayed visible gross lesions or exhibited clinical signs such as weight loss or a rough coat, rather than including healthy animals. This targeted sampling approach may have resulted in an overestimation of the prevalence of infection within the population, as clinically affected animals are more likely to test positive compared to asymptomatic, or subclinically infected individuals.

The results of this study, along with the fact that MAP infection can be present in animals at a young age and in animals considered safe for consumption, are concerning. This is because there is a potential risk for the transmission of MAP to humans through contact with infected boar or by eating undercooked meat or organs that have been contaminated. Although we did not collect information about meat, previous studies showed that MAP can contaminate meat [65,66].

It should be noted that this study only covers a specific region and may not be applicable to other countries on a global scale, which is reflected by the limited number of studies conducted on the prevalence of paratuberculosis in wild boars. However, in regions like Central Portugal, where there are high densities of infected wild boars, the impact on livestock rearing practices can be significant. To control the spread of paratuberculosis in livestock, it would be beneficial to implement routine testing of hunted boars to assess their infection status. This can help mitigate the risk of disease transmission to domestic animals and provide valuable recommendations for control programs.

## 5. Conclusions

This study has provided evidence of MAP infection in wild boar in Portugal and has found that younger boar are particularly susceptible to the disease. The detection of MAP in various organs suggests that the infection can spread throughout the body. Since a large percentage of wild boar are hunted for human consumption, there is a risk of MAP entering the food chain. The zoonotic potential of MAP cannot be ignored, as it is a potential human pathogen. Therefore, it is essential to expand our understanding of MAP infection in wild boar and other wild animals, and to implement measures to control its spread. Further investigations in this area are necessary to obtain a complete understanding of the extent of MAP infections in wildlife.

## Figures and Tables

**Table 1:** Results of culture and PCR in granulomatous lymphadenitis (*n* = 28).

	PCR Negative (N.°; %)	PCR Positive (N.°; %)	Total (N.°; %)
**Culture Negative (N.°; %)**	14 (50.0)	8 (28.6)	22 (78.6)
**Culture Positive (N.°; %)**	1 (3.6)	5 (17.9)	6 (21.4)
**Total**	15 (53.6)	13 (46.4)	28 (100)

Cohen’s k = 0.33; % of agreement = 67.9%.

**Table 2:** Demographic, clinical, and pathological parameters significantly associated with MAP infection.

Demographic, Clinical and Pathological Parameters	N.° Animals	% Infection	*p*	OR	95% IC (OR)
**Age**			0.001		
**Juveniles**	15	86.7		10.16	2.15–48.03
**Subadults and Adults**	82	39.0			
**Lesions in Lungs**			0.028		
**Yes**	17	70.6		3.42	1.10–10.63
**No**	80	41.3			
**Lesions in Mesenteric Lymph Nodes**			0.002		
**Yes**	32	68.8		4.02	1.63–9.92
**No**	65	35.4			
**Positive Ziehl–Neelsen smear in mesenteric lymph nodes**			0.004		
**Yes**	33	66.7		3.57	1.47–8.65
**No**	64	35.9			

## Data Availability

The data presented in this study are available on request from the corresponding author.
